# Individual differences in decision making: Drive and reward responsiveness affect strategic bargaining in economic games

**DOI:** 10.1186/1744-9081-2-35

**Published:** 2006-10-18

**Authors:** Anouk Scheres, Alan G Sanfey

**Affiliations:** 1 Psychology Department, University of Arizona, 1503 E University Blvd., Tucson AZ 85721, USA

## Abstract

**Background:**

In the growing body of literature on economic decision making, the main focus has typically been on explaining aggregate behavior, with little interest in individual differences despite considerable between-subject variability in decision responses. In this study, we were interested in asking to what degree individual differences in fundamental psychological processes can mediate economic decision-making behavior.

**Methods:**

Specifically, we studied a personality dimension that may influence economic decision-making, the Behavioral Activation System, (BAS) which is composed of three components: Reward Responsiveness, Drive, and Fun Seeking. In order to assess economic decision making, we utilized two commonly-used tasks, the Ultimatum Game and Dictator Game. Individual differences in BAS were measured by completion of the BIS/BAS Scales, and correlations between the BAS scales and monetary offers made in the two tasks were computed.

**Results:**

We found that higher scores on BAS Drive and on BAS Reward Responsiveness were associated with a pattern of higher offers on the Ultimatum Game, lower offers on the Dictator Game, and a correspondingly larger discrepancy between Ultimatum Game and Dictator Game offers.

**Conclusion:**

These findings are consistent with an interpretation that high scores on Drive and Reward Responsiveness are associated with a strategy that first seeks to maximize the *likelihood *of reward, and then to maximize the *amount *of reward. More generally, these results suggest that there are additional factors other than empathy, fairness and selfishness that contribute to strategic decision-making.

## Background

For many centuries theoreticians have attempted to build accurate models of how people make judgments, decisions, and choices. To endeavor to answer these important questions about decision-making behavior, researchers from a wide variety of fields have used many different approaches and methods, though for many years this area of interest has been the purview of economists and mathematicians. The resulting mathematical models, such as the family of Expected Utility models [1], were initially proposed as primarily prescriptive, that is, modeling how decisions should be made. However, these models were also taken as good approximations of how people actually make judgments and decisions.

These models remained largely unchallenged as descriptive accounts of behavior until quite recently, when psychological studies of these processes revealed that the models did not in fact capture many of the complexities observed in human decision-making. Careful experiments demonstrated multiple deviations from the "rational" approach of the standard Utility model [2], and new models were proposed that attempted to explain many of these observed decision behaviors. For example, the Prospect theory model of judgment [3] introduces additional parameters to the utility model to account for choices under risk. Further, modern neuroscience techniques have now allowed more biologically plausible models of decision, building upon the proposals from psychology with an increased understanding of the underlying neural substrate [4].

While these psychological and neuroscience models have been unquestionably useful in the study of decision-making, several aspects of this process have thus far been rather understudied. One important question is the degree to which individual differences in fundamental psychological processes can mediate decision-making behavior, in particular economic decision-making.

Models of economic behavior to date, such as the dominant family of Expected Utility models, have typically focused on explaining aggregate behavior (such as that of a market), with little interest paid to individual differences, despite the often considerable variability in decision responses in these situations [2]. Therefore, as a first step it would be useful to examine the degree to which individual differences in certain processes can explain differences in decision-making.

Additionally, these economic models, as noted above, generally have not attempted to understand decision-making in the context of psychological processes, instead concentrating on the decision-maker as a "utility maximizer" with utility typically defined in terms of monetary payoffs. A further goal of this study then, is to demonstrate how differences in psychological processes can help understand decision-making behavior when it deviates from the utility maximization approach.

Experimental studies of economic decision-making have used a wide variety of tasks, often termed "games", in studying this behavior [5]. These tasks, relatively underutilized in psychology and neuroscience, have the advantages that they are typically very simple to understand and implement, have been well-studied, and often produce quite interesting patterns of results. Two games in particular that fulfill these criteria are the Ultimatum Game [6] and the Dictator Game [7]. Both of these games involve the splitting of a sum of money between two players – a Proposer and a Responder – and by using these games in concert, interesting patterns of economic and social decision-making emerge.

In the Ultimatum Game the Proposer is given a sum of money, for example $5, and asked to divide this between themselves and the other player – the Responder. After receiving the Proposer's offer, which can be any division of this amount, the Responder must then either accept or reject the offer. If the Responder accepts, the money is split as proposed. If the Responder rejects, neither player receives any money. Both players are fully aware of the rules of the game and once the decision is made, the game is over.

Game theoretic models prescribe that the optimal solution is for the Proposer to offer as little as possible (say 10 cents), and for the Responder to accept this small amount on the grounds that something is better than nothing [8]. However, in contrast to the prescriptive models, Proposers most commonly offer an even split (i.e. $2.50 to each player in our $5 pot example), and Responders often reject proposals of less than 20% of the total amount (for a summary of recent research see [5]), thus indicating a desire to value equity over monetary reward in some circumstances. The Dictator Game is similar to the Ultimatum Game, except that in this case the Responder cannot reject a proposal and must accept whatever the Proposer gives them. Again, in the Dictator Game both players have full knowledge of the rules and the proposals.

Why do Proposers offer generally fair (i.e. equal split) offers in the Ultimatum Game? There have been two possible explanations suggested. One, people may offer more than a minimal amount to the other player because they care about equity and treating the other player fairly [9]. Two, Proposers may anticipate that Responders will reject too low an offer [10]. So, offering more than a minimal amount may be a strategic decision to increase the likelihood of ending up with at least some amount of money. The Dictator Game has been used in addition to the Ultimatum Game to help tease apart these two potential explanations for higher than minimal offers [11]. Dictator Game offers are made with the certainty that they will be accepted, so giving a 'pure' measure of the degree to which the Proposer cares about equity and fairness, as all strategic considerations should have been removed in this game. Therefore, by examining the pattern of offers made by people in both Ultimatum and Dictator Games, we can make inferences about the motives behind the decisions made by Proposers, and begin to understand to what degree they are influenced by considerations of fairness or by self-interest. Equal splits in both games suggest that the Proposer is motivated by fairness. However, if a Proposer offers an even split in the Ultimatum Game but an unfair offer in the Dictator Game, we can infer strategic motives.

As mentioned above, though these tasks have been widely used in the field of behavioral economics, there have been very few attempts to understand the psychological factors that may underlie decisions made in these situations. Some experimental factors have been studied to date, such as culture [12,13], methodological manipulations [e.g., [14]], description of the games [15], structural changes to the games [e.g., [15-18]], and demographic variables such as gender [19-22], race [20], and age [[23,24]; see 5 for review]. However, we could find only two studies that have explored the role of individual differences in psychological processes in decision making in the Ultimatum Game: one study on selfishness [25], and another on emotions [26]. The goal of this study was therefore to examine whether individual differences in certain personality features are associated with offers made in Ultimatum and Dictator Games, and to what degree these differences influence the various motives underlying the decisions in these contexts.

A personality dimension of obvious relevance to economic decision-making is attitude towards reward. While all modern decision-making models include potential rewards as a key aspect of the utility calculation (e.g. [3]), there is surprisingly little research on how individual sensitivity towards reward may motivate decisions and choices. One of the primary components of the Ultimatum and Dictator Game tasks is the receipt of monetary reward, and therefore we might expect that differential levels of reward sensitivity will be associated with different types of offers in these games.

A psychological measure that has often been used to assess individual differences in reward sensitivity is the Behavioral Activation System (BAS) scale [27]. This measure is generally used in concert with the Behavioral Inhibition System (BIS) scale, which has been designed to measure aversive motivation. As we were interested in the relationship between reward sensitivity and decision-making, we focused solely on the BAS scale and how it might help understand decision-making in the context of these economic games. The BAS scale consists of three sub-scales: (1) the Reward Responsiveness scale measuring positive responses to the occurrence or anticipation of reward; (2) the Drive scale measuring the persistent pursuit of desired goals; and (3) the Fun Seeking scale measuring a willingness to approach a new event on the spur of the moment.

The BAS itself has been defined as a physiological mechanism that underlies appetitive motivation and approach behavior [27-31], and that is sensitive to signals of reward. Dopamine is thought to play an important role in this [32]. Indeed, more recent research has provided evidence for the idea that dopamine plays a crucial role in reward processing [29]. Activity in the BAS is thought to be related to goal-directed behavior and positive feelings when exposed to cues signaling impending rewards [28,29].

Based on this, a simple first-order hypothesis is that higher levels of BAS will be associated with lower offers in both the Ultimatum and Dictator games, reflecting the greater desire for monetary rewards in both games in those with greater sensitivity to rewards. Specifically, we hypothesize that a negative relationship will be observed between offers in these tasks and two of the sub-scales (Reward Responsiveness and Drive), but not in the Fun Seeking scale, because the latter scale assesses more sensation-seeking behavior than reward sensitivity (see [27]).

It is also possible that BAS may have more complex effects on decisions made in the two games, although we do not have specific predictions in this regard. For example, differential effects of BAS on offers made in the two games would reflect a greater influence of reward sensitivity on either strategic or equitable motives in these tasks.

Individual differences in goal-oriented behavior and responsiveness to the anticipation of rewarding outcomes (as measured by the BAS Reward Responsiveness and Drive scales) are potentially key factors behind offers made in the two tasks of interest. Therefore, finding a relationship between these scales and proposal amounts would help in understanding the psychological motivations behind economic decisions. This study therefore examined performance in the Ultimatum and Dictator Games, and investigated the contribution of the reward system to decision-making in these tasks.

## Methods

### Participants

Subjects were 69 undergraduate students at the University of Arizona. Two participants were excluded from data analyses (see below). Therefore, we describe here data from 67 participants. This sample consisted of 31 males and 36 females. The age range of participants was 18–29, with a mean age of was 19.7 (*SD *= 2.3), and all signed informed consent forms before participation.

### BIS/BAS Rating Scale

All participants completed the BIS/BAS scales [27]. The BAS Reward Responsiveness scale has 5 items pertaining to positive responses to the anticipation or occurrence of reward. The BAS Drive scale includes 4 items that focus on the pursuit of desired goals. The BAS Fun Seeking scale has 4 items focusing on a desire for new rewards and a willingness to approach a potentially rewarding event on the spur of the moment. Responses were made on a 4-point scale, with 1 indicating strong disagreement and 4 indicating strong agreement. The internal reliabilities of the BAS scales are generally high, and the test-retest reliabilities are moderate. Convergent, discriminant, and predictive validity of the scales are good [27].

### Decision Making Tasks

Participants played two decision-making tasks. The first one was the Ultimatum Game. All participants were run simultaneously in one session, and first played the Ultimatum Game as Proposer. They were instructed to distribute $5 between themselves and their randomly assigned, anonymous, partner. They were told that their partner could either accept or reject their offer. If the offer was accepted, the money would be divided as proposed. If the offer was rejected, neither player would receive anything. It was emphasized that this game was played for real money and that participants would be paid directly based on the decisions made in the games. The Proposer was given a slip of paper with two rows indicating amounts displayed in fifty-cent increments from $0 to $5, and asked to circle the amount they wished to keep for themselves and the corresponding amount they wished to offer to their partner. After the Proposer had indicated their offer, the slip of paper was returned to the experimenters. The experimenters then gave the slip to a randomly chosen Responder, with the restriction that nobody received their own offer. Following this, participants were informed that they would now play the game as Responders and that they had the opportunity to accept or reject the anonymous offer made to them.

In order to make sure that task administration with this large group of participants was done in an efficient manner, all participants were given a laminated card with a code (a random number between 1 and 70). Prior to the experiment, the experimenters had randomly paired code numbers, with the restriction that a code number could not be paired with itself. On beginning the experiment, participants were given a slip of paper on which to write their offers. These slips also contained the code of both the Proposer (that participant) and the Responder (whose identity was unknown to the Proposer). After the offers were made, the slips were collected and redistributed to the pre-assigned Responder.

Next, participants then played the Dictator Game. The procedure was the same as outlined above, except that in this game the Responder did not have the option to reject the offer, and had to accept whatever the Proposer offered. Proposers made dictator offers to a different, again anonymous, partner than in the Ultimatum Game.

After task completion, the experimenters calculated participants' payoffs for the tasks, and all participants completed the BIS/BAS Rating Scale. When participants had completed the rating scales, they handed these in to one of the three experimenters, who then informed them if the offer they made in the Ultimatum Game was accepted or rejected, and how much was given to them by their partner in the Dictator Game. They were then paid accordingly.

The following dependent variables were used for statistical analyses: Ultimatum Game proposals, Dictator Game proposals, and difference scores (Ultimatum Game proposal minus Dictator Game proposals). Using the two games in combination, as reflected by the difference score, helped us to asses the extent to which a "fairness component" and a "strategic component" contributed to the offers that were made. In the Ultimatum Game, people may offer more than a minimal amount because they care about equity (fairness component), or because they anticipate that Responders will reject too low an offer (strategic component). The larger the discrepancy between Ultimatum Game proposal and Dictator Game proposal, the larger the strategic component in making offers. The smaller the discrepancy between Ultimatum Game proposal and Dictator Game proposal, the smaller the strategic component in making offers (see also Statistical Analyses).

### Statistical Analyses

In order to test the hypothesis that individual differences in the BAS are associated with decision making in economic games, we computed bivariate correlations between the BAS scales and offers in the Ultimatum Game and Dictator Game. Because Ultimatum Game offers correlated with Dictator Game offers (*r *= .28, *p *< .05), we also computed partial correlations between BAS scale scores and Ultimatum Game offers (controlling for Dictator Game offers), and between BAS scale scores and Dictator Game offers (controlling for Ultimatum Game offers). Partial correlations clarified whether Dictator Game offers and Ultimatum Game offers were uniquely correlated with BAS scales, over and beyond due to shared variance between the two games. Finally, in order to understand to what extent correlations between BAS scales and offers in both games were associated with strategic or fairness-related considerations, we computed correlations between BAS scales and difference scores (Ultimatum Game offers minus Dictator Game offers).

### Outliers

Data for two participants were excluded from the analyses because they showed a highly unusual pattern of responding [5], offering more than half to their partners in both games ($3.50 and $4.50 respectively). Analyses including these outliers did not change the results.

## Results

### Descriptives

In our participants, mean total scale scores for the BAS scales were very similar to those reported in the original sample [23]: BAS Reward Responsiveness 17.4 (*SD *2.0), BAS Drive 11.1 (*SD *2.3), BAS Fun Seeking 12.6 (*SD *2.1). No differences between males and females were observed for any of the BAS scales (all *p*'s > .65).

The mean Ultimatum Game proposal was $2.37 (47% of $5) (*SD *= .43), with $2.50 being the mode, very similar to that generally observed in these studies [5]. The mean Dictator Game proposal was $1.21 (24%) (*SD *= 1.1), with a mode of $0. Seven percent of all Ultimatum Game offers were quite low ($1.50 or less). Of those five Ultimatum Game offers, two were rejected (40%). Ninety-three percent of all Ultimatum Game offers were $2 or more. Of those 62 offers, one was rejected (1.6%). The mean of the rejected offers was $1.33 (*SD *1.0), as compared to a mean of $2.45 (*SD *.49) for accepted offers. No gender differences were found for Ultimatum Game proposals (*F*(1,66) = .80, *ns*) or Dictator Game proposals (*F*(1,66) = .22, *ns*).

Because the Dictator Game was always played after the Ultimatum Game, one might expect that received Ultimatum Game offers may affect proposals made in the Dictator Game. In order to test for this potential order effect we computed correlations between received Ultimatum Game proposals and offers made in the Dictator Game. There was no significant correlation (*r *= -.05, *ns*).

### Correlational analyses: Ultimatum Game

When computing bivariate correlations, offers made in the Ultimatum Game did not correlate significantly with any of the BAS scales (Table 1). However, after controlling for proposals made in the Dictator Game, Ultimatum Game proposals did correlate positively with the BAS Drive scale, but not significantly with the BAS Reward Responsiveness and Fun Seeking scales (Table 1).

**Table 1:** Bivariate and partial correlations between proposals made and the Behavioral Activation System scales

	UG proposal	UG proposal controlled for DG proposal	DG proposal	DG proposal controlled for UG proposal	UG-DG proposal	BAS RR	BAS Dr	BAS FS
BAS RR	.08	.21	-.34**	-.37**	.38***	1	-	-
BAS Dr	.18	.27*	-.27*	-.34**	.36**	.55***	1	-
BAS FS	-.01	.03	-.15	-.14	.15	.51***	.45***	1

UG, Ultimatum Game; DG, Dictator Game; BAS, Behavioral Activation System; RR, Reward Responsiveness; Dr, Drive; FS, Fun Seeking.

*p < .05; **p < .01; ***p < .001

### Correlational analyses: Dictator Game

Offers made in the Dictator Game correlated negatively and significantly with BAS Reward Responsiveness and BAS Drive, but not with BAS Fun Seeking. When controlling for Ultimatum Game proposals, Dictator Game proposals still correlated negatively with the BAS Drive scale, and even more strongly with the BAS Reward Responsiveness scale. No correlation with Fun Seeking was found (Table 1).

### Correlational analyses: Difference Scores

**Figure 1:**
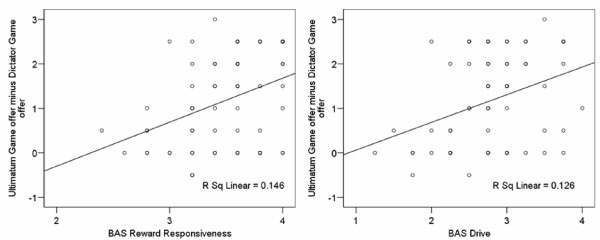
Correlations between difference scores (Ultimatum Game offer minus Dictator Game offer) and average Behavioral Activation System Reward Responsiveness Scale (left plot) and Drive Scale (right plot) scores.

Difference scores (Ultimatum Game proposals minus Dictator Game proposals) correlated negatively and significantly with the BAS Reward Responsiveness scale and the BAS Drive scale (Figure 1). No correlation between difference scores and BAS Fun Seeking was found (Table 1).

## Discussion

This study investigated whether individual differences in the BAS, namely Reward Responsiveness, Drive, and Fun Seeking, were associated with offers made in two decision-making tasks: the Ultimatum Game and the Dictator Game. We found that higher scores on the BAS Drive scale were associated with a pattern of higher offers on the Ultimatum Game (after controlling for Dictator Game offers), lower offers on the Dictator Game, and a correspondingly larger discrepancy between Ultimatum Game and Dictator Game offers. Higher scores on the BAS Reward Responsiveness scale were also associated with lower offers on the Dictator Game, and a larger discrepancy between Ultimatum Game and Dictator Game offers, although the correlation with Ultimatum Game offers fell short of significance. The BAS Fun Seeking scale did not correlate with offers made in these decision making tasks.

These findings demonstrate that individual differences in basic psychological processes, such as reward sensitivity, can help understand performance on economic and social decision-making tasks, processes which hitherto have not been used to explain behavior in these contexts. Our results provide evidence for the hypothesis that individual differences in certain personality features, in this case Drive and Reward Responsiveness, contribute to individual differences in decision making, in this case offers made in the Ultimatum and Dictator Games. We had hypothesized that higher BAS scores would be associated with lower offers on both the Ultimatum Game and the Dictator Game, on the assumption that individuals with higher reward sensitivity would attempt to maximize their monetary earnings on each trial. As expected, higher scores on BAS Drive and Reward Responsiveness were associated with lower offers on the Dictator Game. However, contrary to our predictions, higher BAS Drive and Reward Responsiveness scores were associated with *higher *offers on the Ultimatum Game. Moreover, higher BAS scores were associated with larger drops from Ultimatum Game to Dictator Game offers.

This pattern of correlations is best interpreted, we believe, by proposing that individuals with high scores on Reward Responsiveness and Drive scales use particular strategies in decision-making. Specifically, this strategy first seeks to maximize the *likelihood *of reward, and then seeks to maximize the *amount *of reward. In the Ultimatum Game, when offers can be rejected by their partner – thus leaving both players with nothing – people with higher scores on Drive and Reward Responsiveness make higher offers. While in isolation it may appear that higher Ultimatum Game offers may reflect increased concern for equity and fairness, the pattern of results suggests that instead it seems designed to ensure that the offer will not be rejected, thus resulting in a higher probability of getting something instead of nothing. In the Dictator Game, when the partner cannot reject the offer, people with higher Reward Responsiveness and Drive now make lower offers, thus maximizing their own gains. The difference score between Ultimatum Game and Dictator Game offers directly reflects the strategic component (as opposed to the fairness component) of the offers made. Thus, higher scores on Reward Responsiveness and Drive are associated with lower offers to the partner, but only when there is the certainty of this offer being accepted. When the offer can be rejected Reward Responsiveness and Drive are associated with *higher *offers, potentially as a strategy to ensure to obtain something, rather than running the risk of obtaining nothing.

These results indicate that there are additional factors other than empathy, fairness and selfishness that determine whether a person offers a larger or smaller piece of the pie to their partner [[34]; see also 25]. Specifically, these results offer insight into individual differences that contribute to strategic decision making in Ultimatum Game and Dictator Game: Drive and Reward Responsiveness are associated with the strategic component of decision making, as reflected in the differences between Ultimatum Game and Dictator Game offers.

Use of both Ultimatum Game and Dictator Game games in the current study allowed us to determine more precisely the motivations behind the offers in these tasks. As discussed previously, Dictator Game offers are a relatively "pure" measure of altruism, while Ultimatum Game offers typically mix equity and strategy. In a previous study [25], the relationship between Ultimatum Game offers and personality measures (in this case extraversion) was investigated. A positive correlation between Ultimatum Game offers and individual differences in extraversion was found, and interpreted as indicating increased equity considerations on the part of proposers with higher extraversion scores. However, if that study had also included the Dictator Game, the authors may have reached a different conclusion. In our dataset, we similarly find a positive correlation between Ultimatum Game offers and the personality measure used here (BAS Reward Responsiveness). However, the negative correlation found between this individual difference measure and Dictator Game offers allows us to draw a fuller conclusion about the nature of decisions in these games, namely that both of these correlations can be explained by increased desire for certainty of reward in individuals with greater Reward Responsiveness. Therefore, by using different games in concert we can make, we believe, more detailed conclusions about the motivations behind the decisions.

The lack of correlation between offers and BAS Fun Seeking is in line with our expectations. Given that the Fun Seeking scale measures sensation seeking and acting on the spur of a moment (e.g., "I crave excitement and new sensations", "I often act on the spur of a moment") more than reward sensitivity, we did not expect this scale to correlate with offers in economic games. Thus, only specific aspects of BAS, in this case Reward Responsiveness and Drive, correlated with decision making in economic games.

Given the substantial correlations between the 3 BAS scales (Table 1), one question is whether BAS Reward Responsiveness and Drive *uniquely *contribute to Ultimatum Game and Dictator Game offers. To address this question, we computed partial correlations between the BAS Drive scale and the Ultimatum Game-Dictator Game difference score, and between the BAS Reward Responsiveness scale and the Ultimatum Game-Dictator Game difference score, while controlling for the other 2 BAS scales. This analysis showed that only the correlation between Reward Responsiveness and the Ultimatum Game-Dictator Game difference score remained significant (*r *= .26, *p *< .05), while the correlation between Drive and Ultimatum Game-Dictator Game difference score fell short of significance (*r *= .21, *p *< .10). This suggests that the use of a reward-maximizing strategy in the Ultimatum Game and Dictator Game is more strongly associated with Reward Responsiveness than with Drive.

This study focused on the role of personality features, in particular various aspects of BAS, in relation to offers made in the Ultimatum Game and Dictator Game. Future research will also need to examine the potential role of these personality features in the Responders' reactions of accepting or rejecting Ultimatum Game proposals made to them. The current dataset did not allow for such an analysis, because, as is usually observed, most offers in the Ultimatum Game were fair and therefore accepted by the responders.

Additionally, future research would benefit from using brain imaging techniques to increase our understanding of the neural basis of individual differences in the reward system and how these contribute to decision making in economic games. One fMRI study has investigated the neural basis of rejections versus acceptances in the Ultimatum Game [35]. However, no research to date has focused on the neural basis of proposals made. Individual differences in self-reported Reward Responsiveness and Drive are likely associated with individual differences in brain activation in areas previously shown to be important in reward processing, such as ventral striatum and orbitofrontal and medial prefrontal areas [36]. In a recent brain imaging study, individual differences in extraversion appeared to account for a substantial proportion of variance in reward-related activation in bilateral medial orbitofrontal cortex and right nucleus accumbens [37]. Future brain imaging research can shed light on how individual differences in the brain reward system contribute to strategic decision making in the Ultimatum and Dictator Games.

## Conclusion

This experiment showed that individual differences in Reward Responsiveness and Drive were linked to strategic decision-making in both the Ultimatum and Dictator Games, two commonly-used economic decision tasks. More broadly, these results demonstrate that investigating the role of psychological processes in these type of tasks can help gain a better understanding of the motives that underlie decision-making.

## Competing interests

The author(s) declare that they have no competing interests.

## Authors' contributions

AS designed the study, carried it out, analyzed the data and drafted the manuscript. AGS participated in designing the study and drafting the manuscript. Both authors read and approved the final manuscript.
